# The endotoxin hypothesis of Alzheimer’s disease

**DOI:** 10.1186/s13024-024-00722-y

**Published:** 2024-04-01

**Authors:** Guy C. Brown, Michael T. Heneka

**Affiliations:** 1 Department of Biochemistry, University of Cambridge, Cambridge, United Kingdom; 2 Luxembourg Centre for Systems Biomedicine, University of Luxembourg, Belvaux, Luxembourg

**Keywords:** Lipopolysaccharide, Alzheimer’s disease, Endotoxin, Inflammation, Microglia, Gut, Neurodegeneration, Neuroinflammation

## Abstract

Lipopolysaccharide (LPS) constitutes much of the surface of Gram-negative bacteria, and if LPS enters the human body or brain can induce inflammation and act as an endotoxin. We outline the hypothesis here that LPS may contribute to the pathophysiology of Alzheimer’s disease (AD) via peripheral infections or gut dysfunction elevating LPS levels in blood and brain, which promotes: amyloid pathology, tau pathology and microglial activation, contributing to the neurodegeneration of AD. The evidence supporting this hypothesis includes: i) blood and brain levels of LPS are elevated in AD patients, ii) AD risk factors increase LPS levels or response, iii) LPS induces Aβ expression, aggregation, inflammation and neurotoxicity, iv) LPS induces TAU phosphorylation, aggregation and spreading, v) LPS induces microglial priming, activation and neurotoxicity, and vi) blood LPS induces loss of synapses, neurons and memory in AD mouse models, and cognitive dysfunction in humans. However, to test the hypothesis, it is necessary to test whether reducing blood LPS reduces AD risk or progression. If the LPS endotoxin hypothesis is correct, then treatments might include: reducing infections, changing gut microbiome, reducing leaky gut, decreasing blood LPS, or blocking LPS response.

## Background

Alzheimer’s disease (AD) is a progressive neurodegenerative disease, primarily affecting memory and thinking, but also associated with depression, anxiety, personality change, spatial/visual disturbance and poor judgement (Scheltens et al. 2021). The Alzheimer’s brain is characterised by: i) amyloid plaques, mainly consisting of extracellular aggregates of amyloid beta (Aβ, ii) TAU tangles, mainly consisting of intraneuronal aggregate of hyperphosphorylated TAU, iii) neuroinflammation, including activated microglia, and iv) synaptic loss, neuronal loss and brain atrophy (Scheltens et al. 2021). AD is one of the main causes of dementia, morbidity and death in the world, and its prevalence is increasing due to ageing populations (GBD 2022). Most (>90%) AD patients suffer from late-onset Alzheimer’s disease (LOAD) diagnosed after the age of 60 years, and with a prevalence doubling every subsequent 8 year of remaining life. Whereas, early-onset AD is AD diagnosed before the age of 60. LOAD is normally preceded by mild cognitive impairment (MCI) in which memory and thinking are impaired, but the individual is still capable of independent living (Scheltens et al. 2021).

**Fig. 1:**
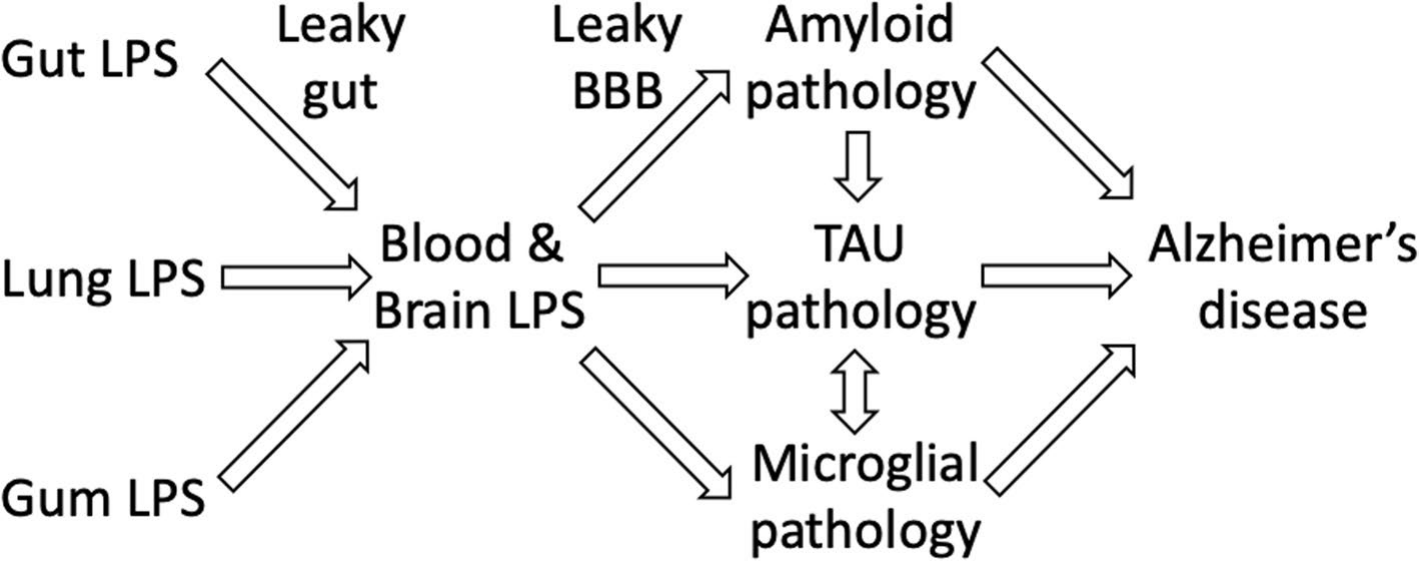
Summary of the endotoxin hypothesis of Alzheimer’s disease. LPS from gut, lungs or gums may increase blood LPS, which may directly or indirectly increase the pathology of Aβ, TAU and microglia, to exacerbate Alzheimer’s disease

AD patients have higher than normal levels of LPS endotoxin in their blood (Zhang et al. 2009; Loffredo et al. 2020; Andreadou et al. 2021; Sánchez-Tapia et al. 2023), and when these elevated levels of LPS are injected into the blood of health humans, they induce cognitive dysfunction (Bahador & Cross 2007; Sandiego et al. 2015). In mice, LPS exacerbates amyloidopathy and tauopathy by multiple mechanisms. Thus, it is possible that endotoxin contributes to AD, and this has been suggested by various researchers (Zhan & 2018; Brown 2019; Kalyan et al. 2022). Here, we make an explicit and specific statement of the endotoxin hypothesis of AD (summarised in Fig. 1), and gather evidence for and against it, so that the hypothesis can be more robustly tested.

### Endotoxin and its toxicity

Endotoxins are components of bacteria that are toxic to mammals. One of the main endotoxins is lipopolysaccharide (LPS), and the term ‘endotoxin’ is often used synonymously with LPS. The LPS molecule consists of lipid A (between 4 and 6 fatty acids linked to a phosphorylated disaccharide), attached to the “core” (a short sugar chain with various modifications), attached the O-antigen (a long linear chain of sugars of very variable length) (Raetz & Whitfield 2002). LPS is produced by Gram-negative bacteria, constitutes much of the surface of the outer cell membrane of such bacteria, and can be released as vesicles from live or dead bacteria (Raetz & Whitfield 2002). The main source of endotoxin in healthy humans is gut-resident Gram-negative bacteria, including *Bacteroides fragilis* and *Escherichia coli*. However, bacterial infections may bring new sources, for example, gum disease results in LPS from *Porphyromonas gingivalis* in blood and brain (Fu Y et al. 2023). Different species of Gram-negative bacteria produce somewhat different lipopolysaccharides with different capacity to induce inflammation, for example the LPS of *Escherichia coli* and *Porphyromonas gingivalis* is pro-inflammatory, whereas the LPS of *Bacteroides fragilis* is normally anti-inflammatory by binding to Toll-like receptor 4 (TLR4) but not activating it (Vatanen et al. 2016). Although, *Bacteroides fragilis* can also produce a potently pro-inflammatory form of LPS (Lukiw 2016; Zhao et al. 2022).

Even low levels of LPS induce a strong inflammatory response when injected into mammals, mainly via binding TLR4 and its co-receptor myeloid differentiation factor 2 (MD2) on the surface of immune cells, resulting in NF-κB transcriptional activation of hundreds of inflammatory genes, including pro-inflammatory cytokines such as tumor necrosis factor α (TNF-α), interleukin 6 (IL-6) and IL-1β (Bryant et al. 2010). Intracellular LPS can directly activate human caspase-4 or -5, which can cleave and activate caspase-1, which can cleave and activate IL-1β (Pfalzgraff et al., 2019). Active caspase-1 can also cleave and activate gasdermin D, which permeabilises the plasma membrane allowing IL-1β out, but may also kill immune cells by pyroptosis (Kayagaki et al. 2015; Shi et al. 2015). LPS can also activate complement and complement receptor 3 (Wright et al. 1989), and scavenger receptors, such as scavenger receptor A (SR-A) and macrophage receptor with collagenous structure (MARCO) (Hampton et al. 1991).

Humans are uniquely sensitive to endotoxin, for example, intravenous injection of 100 ng LPS induces inflammation in body and brain, and >1 mg endotoxin can result in death (Bahador & Cross 2007). And this is much lower than the amount of endotoxin found in the gut: roughly 1g in the Gram-negative bacteria of the gut lumen (Sender et al. 2016). Thus, a ‘leaky gut’ can increase plasma endotoxin levels, resulting in ‘endotoxemia’ (Camilleri 2019; Mohammad & Thiemermann 2021). Intravenous injection of 1 ng LPS/kg into healthy human volunteers caused: increased blood cytokines, sickness behaviour and microglial activation for 1-3 hours (Sandiego et al. 2015).

The response to endotoxin depends strongly on the dose and time course of exposure, partly because prior exposure to endotoxin normally desensitises cells to subsequent endotoxin exposure (endotoxin tolerance) (Bahador & Cross 2007). Because low-dose endotoxin can induce an insensitive or anti-inflammatory state, it can be neuroprotective in certain conditions, and has been suggested as treatment (Mizobuchi & Soma 2021). However, very low levels of endotoxin can also sensitise cells to subsequent endotoxin exposure (endotoxin priming) (Bahador & Cross 2007; Neher & Cunningham 2019). Thus, concentration and time course of endotoxin exposure are important to response. Rodents and all other laboratory mammals are much less sensitive to endotoxin than humans (Bahador & Cross 2007) making them less than ideal models of endotoxin response in humans (Seok et al. 2013).

### Endotoxin levels are increased in blood and brain of AD patients

Blood plasma or serum levels of LPS have been reported by five different research groups to be 1.5 to 7 fold higher in AD patients than age-matched controls (Table 1). LPS can be measured by a limulus amebocyte lysate (LAL) assay (which measures LPS activity in inducing blood clotting) or by a sandwich ELISA (which measures antibody binding to LPS). Pei et al. 2023 also reported significantly increased blood LPS in AD patients, but they did not say how LPS was measured and whether it was measured in serum or plasma, so their results are not included in Table 1. Measuring LPS in healthy human blood can be technically challenging, and most LAL and many ELISA kits are insufficiently sensitive to measure the low levels of LPS in healthy human plasma or serum (Hurley 1995; Gnauck et al. 2016). Also, different ELISA kits may be detecting different species of LPS, depending on the anti-LPS antibodies used, which raises the question of which species of LPS should be being measured. Never-the-less, where both LAL and ELISA kits were used to measure blood LPS levels, a quantitatively similar conclusion was reached: that LPS levels were twice as high in AD patients as age-matched controls (Loffredo et al. 2020). Note that the absolute levels of LPS in control serum were much higher in Sánchez-Tapia et al. 2023 than other studies, probably due to the different ELISA test using different antibodies with different sensitivities to different LPSs, but this does not invalidate the finding that the relative levels of detected LPS were higher in AD serum than control serum.

**Table 1:** Endotoxin levels in blood of AD patients and age-matched controls

**Study**	**LPS in AD**	**LPS in controls**	**LAL or ELISA** **Plasma or Serum**
Zhang et al. 2009	60 ± 12 pg/ml, mean ± SEM: *N* =18	20 ± 2 pg/ml, mean ± SEM: *N*=18	LAL Plasma
Loffredo et al. 2020	26 ± 9 pg/ml, mean ± 95% CL *N*=47	12 ± 6 pg/ml, mean ± 95% CL *N*=64	ELISA and LAL Serum
Andreadou et al. 2021	97 ± 55 AU, mean ± SD mean ± SD *N*=18	14 ± 18 AU, *N*=13	ELISA Serum
Marizzoni et al. 2023	0.3 ± 0.05 AU, median ± 95% CL *N* =34	0.2 ± 0.1 AU median ± 95% CL *N*=13	ELISA Plasma
Sánchez-Tapia et al. 2023	694 ± 100 ng/mL, mean ± SD *N*=13	157 ± 100, mean ± SD *N*=42	ELISA Serum

The first column gives the reference for the study, the second and third columns give the measured level of LPS in AD patients and age-matched controls, where N is the number of such people measured, and the fourth column gives the method of LPS measurement (*LAL* Limulus amebocyte lysate assay, *ELISA* Enzyme-linked immunosorbent assay) and whether LPS was measured in blood serum or plasma

*AU* Arbitrary units, *SEM* Standard error of mean, *SD* Standard devaiation, *CL* Confidence limits

**Fig. 2:**
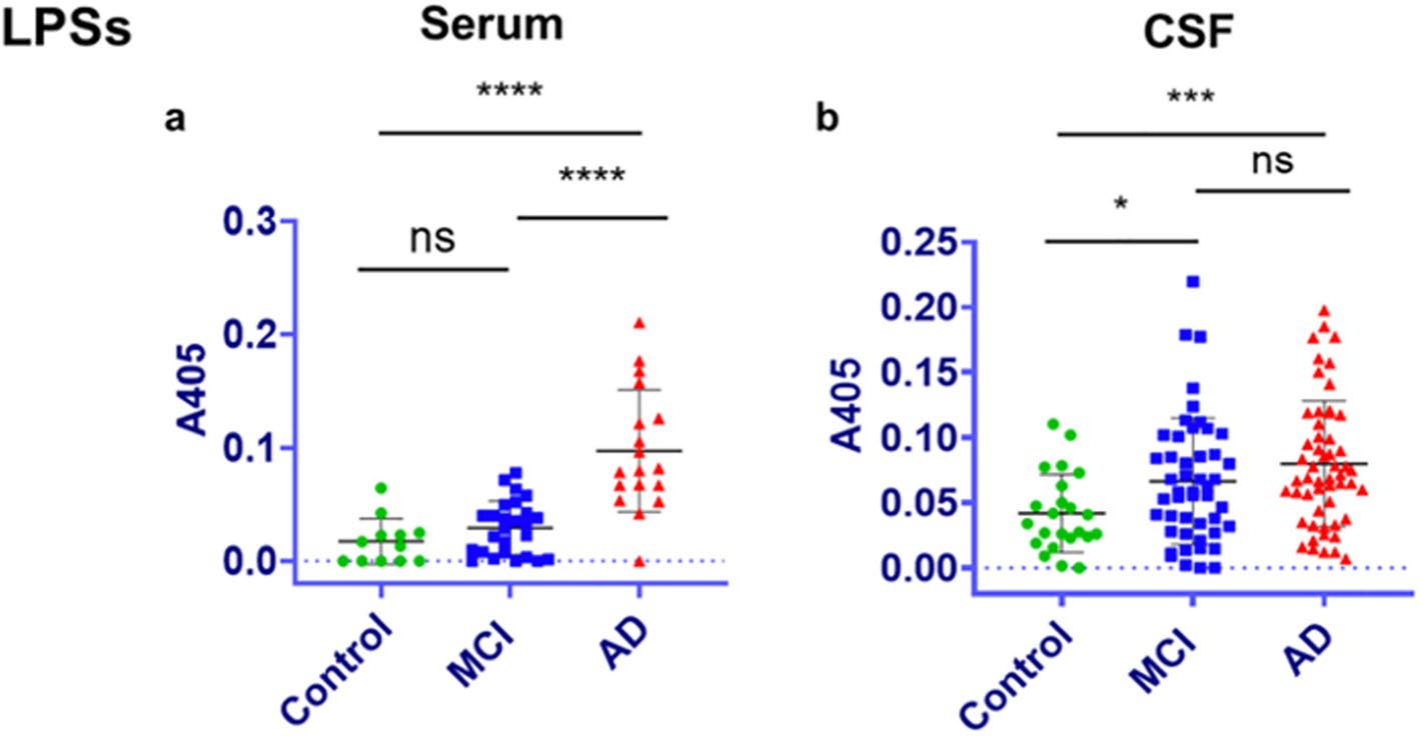
Serum and CSF (cerebralspinal fluid) measurements of LPS endotoxin by sandwich ELISA in AD and MCI patients and age-matched controls. Reprinted from Andreadou EG et al. 2021, with permission from Elsevier. A405 is the absorbance measurement proportional to the amount of LPS

Although the mean blood plasma levels of LPS are clearly higher in AD patients than age-matched controls, the distribution of endotoxin levels in different people indicates that a proportion of AD patients have endotoxin levels overlapping with those of controls (Fig. 2). Thus, endotoxin is not elevated in all AD patients – however, it remains possible that these AD patients had elevated endotoxin (days, months or decades) prior to the measurement – and transiently elevated LPS levels may have long-lasting effects (Cao et al. 2021). Note, that plasma endotoxin levels are also elevated in Parkinson’s disease, amyotrophic lateral sclerosis and other pathologies (Zhang et al. 2009; Loffredo et al. 2020), so elevated endotoxin is not specific to AD, suggesting that elevated endotoxin is not sufficient to induce AD.

A cross-sectional analysis of 127 patients without dementia in a memory clinic (average age 76) revealed plasma LPS concentration was associated with mild cognitive impairment, and that high plasma endotoxin levels was associated with a more rapid cognitive decline (Saji et al. 2022). Sánchez-Tapia et al. 2023 reported that serum LPS levels were higher in MCI patients (399 ± 150, *N*=32) than aged-matched controls (157 ± 100, *N*=42) measured by ELISA. Sánchez-Tapia et al. 2023 also reported that serum LPS levels correlated with memory dysfunction in MCI and dementia patients. Andreadou et al. 2021 found a higher mean level of serum LPS in MCI patients than controls, but this was not significant (Fig. 2). Andreadou et al. 2021 also found that serum endotoxin levels correlated with CSF levels of Aβ_1-42_ and TAU levels, as well as memory impairment in MCI and AD patients.

The reason that endotoxin levels are elevated in AD patients is unclear. Possibilities include: a dysregulated gut microbiome, a leaky gut or bacterial infections. AD patients have increased infections by specific microbes, including: i) pneumonia, induced by lung infection by *Chlamydia pneumoniae*, and ii) gum disease, induced by gum infection by *Porphyromonas gingivalis* (Fu et al. 2023). Both *Chlamydia pneumoniae* and *Porphyromonas gingivalis* are Gram-negative bacteria and intracellular parasites of mammalian cells, infections of which are associated with AD (Chacko et al. 2022; Fu et al. 2023), and there is limited evidence of their presence in the brains of AD patients (Balin et al. 1998; Gérard et al. 2006; Poole et al. 2013; Dominy et al. 2019). Thus, these infections are potential sources of increased LPS in the body and brains of AD patients (along with gut bacteria).

There have been several published studies of gut microbiome changes in AD. Cattaneo et al. (2017) reported higher *Escherichia* and *Shigella* species (both Gram negative) and lower *Eubacterium* species (Gram positive) in AD relative to controls. Vogt et al. (2017) reported higher *Bacteroidetes* (Gram negative, but relatively anti-inflammatory) and lower Firmicutes and *Bifidobacterium* species (both Gram positive) in AD patients. Zhuang et al. (2018) reported higher *Actinobacteria* (Gram positive) and lower *Bacteroidetes* (Gram negative, but relatively anti-inflammatory) in AD. Liu et al. (2019) reported higher *Proteobacteria* (Gram negative) and lower *Firmicutes* species (Gram positive) in AD. Marizzoni & 2023 reported an increase in gut *Clostridia*_UCG-014 in AD. These studies are not consistent about the bacterial species changes in AD, however, most studies report an increase in pro-inflammatory LPS-producing species in the guts of AD patients. On the other hand, low levels of anti-inflammatory *Faecalibacterium prausnitzii* in the gut of patients correlate with high levels of LPS in serum (Sánchez-Tapia et al. 2023; Pei et al. 2023).

Brain levels of LPS have also been reported to be increased in AD as measured by immunocytochemistry and western blot (Zhan et al. 2016, 2018; Zhao et al. 2017; Zhao, Jaber & Lukiw 2017, and Zhao et al. 2022). Similarly, Andreadou et al. (2021) reported that the endotoxin levels of CSF were higher in AD patients than non-demented, age-matched controls, measured by ELISA (Fig. 2). Gram-negative bacteria of the nasal and oral cavities have been found in AD brains, but not control brains, including *Chlamydia pneumoniae* (Balin et al. 1998), *Borrelia burgdorferi* (Miklossy 2011b), and *Porphyromonas gingivalis* (Dominy et al. 2019), suggesting the possibility that brain LPS in AD patients may be derived from bacterial infections of the brain that are not clinically apparent.

### Genetic and non-genetic risk factors for AD affect endotoxin levels or response

LOAD is approximately 2/3 genetic and 1/3 environmental (Gatz et al. 2006). Variants of genes encoding amyloid precursor protein (APP) and the presenilin’s (PSEN1, and PSEN2, which generate amyloid beta) cause early-onset AD, but have relatively little effect on LOAD (Bellenguez et al. 2022). Mutations of MAPT, the gene encoding TAU, cause primary tauopathies such as progressive supranuclear palsy (PSP), but are not associated with secondary tauopathies such as AD, i.e. AD patients do develop tauopathy, but without obligatory MAPT mutations (Bellenguez et al. 2022). Many of the genes that do associate with LOAD risk affect innate immunity (Bellenguez C et al. 2022), and thus might be compatible with the endotoxin hypothesis of AD, either because the gene product directly interacts with LPS (APOE and TREM2) or because the gene product affects LPS response or susceptibility to infection.

The main genetic risk for AD is APOE isoform: APOE4 increasing risk, APOE2 decreasing risk and APOE3 being neutral. LPS strongly induces ApoE expression in mice, which then binds to LPS (Petruk et al. 2021) and redirects it from macrophages to liver cells, enabling the beneficial clearance of LPS from blood (Rensen et al. 1997; Van Oosten et al. 2001). Humans with the APOE4 variant are more sensitive to injected LPS than those with APOE3, and similarly mice with endogenous ApoE replaced with APOE4 are more sensitive to LPS than those replaced with APOE3 (Vitak et al. 2009; Gale et al. 2014), consistent with APOE4 being detrimental in AD by increasing LPS toxicity or decreasing LPS clearance. APOE can bind and kill Gram-negative bacteria (Petruk et al. 2021), and if APOE4 does this less efficiently, this might lead to more Gram-negative infections, and thereby higher LPS, but this has not been shown.

The other genes most strongly associated with AD risk are *BIN1*, *CLU*, *TREM2* and *CR1* (Bellenguez et al. 2022). Of these, BIN1 is known to strongly affect the inflammatory response of microglia to endotoxin (Sudwarts et al. 2022). Endotoxin induces the expression and release of both clusterin (ApoJ) and ApoE by glial cells (Saura et al. 2003). Macrophages from clusterin knockout mice have more of an M1 inflammatory response to endotoxin (Weng et al. 2021). TREM2 can bind to endotoxin (Daws et al. 2003) and regulate the inflammatory response of macrophages and microglia to endotoxin (Gao et al. 2013; Wang et al. 2019). CR1 regulates complement activation by endotoxin (Lachmann et al. 2016). Thus, the genetics of AD are compatible with the endotoxin theory of AD. In addition, as many different AD risk genes affect innate immunity, it is possible that these genes affect the probability of bacterial infection and thereby LPS levels in the body. For example, ApoE binds and is toxic to Gram-negative bacteria (Petruk et al. 2021).

The main non-genetic factors affecting AD risk is age - LOAD risk doubles every 8 years over the age of 60. And age is known to increase the levels and response to LPS in humans - blood LPS levels increased several fold with age (Sánchez-Tapia et al. 2023). Aging is also associated with an increased acute-phase response to LPS injection, including initial hyper-reactivity, prolonged inflammatory activity, and prolonged fever response (Krabbe et al. 2001; Bahador & Cross 2007). Thus, the main non-genetic risk factor for AD is also compatible with the endotoxin hypothesis of AD.

Environmental factors that affect AD risk include infections. Hospitalization for common bacterial infections (pneumonia, gingivitis, urinary tract infections, *Helicobacter pylori*) is associated with increased subsequent dementia risk (Ehlenbach et al. 2010; Bu et al. 2015; Frölich et al. 2020; Shindler-Itskovitch et al. 2016). For example, Tate et al. (2014) found that hospitalisation with pneumonia (associated with Gram-negative *Chlamydia pneumoniae*) increased the risk of subsequently developing dementia within 3 years after the hospitalisation. Gum disease caused by Gram-negative *Porphyromonas gingivalis* also increases AD risk (Ishida et al. 2017; Ding et al. 2018; Fu et al. 2023). Thus, infection with Gram-negative bacteria is associated with increased AD risk, consistent with LPS promoting AD.

A prospective study of 12,000 middle-aged people found that raised blood markers of systemic inflammation was associated with a more rapid decline in cognition over a subsequent 20-year period, i.e. systemic inflammation appears to promote subsequent cognitive decline (Walker et al. 2019). As blood LPS can cause systemic inflammation, this suggests the possibility that: i) raised blood LPS causes subsequent cognitive decline by inducing systemic inflammation, and/or ii) raised blood LPS may be one cause of systemic inflammation observed in this population. Systemic inflammation is one of the main causes of delirium, and delirium is a risk factor for subsequent dementia, and delirium increases the rate of cognitive decline in those with dementia (Fong et al. 2022).

Obesity, diabetes, cardiovascular disease and poor diet are also non-genetic risk factors for AD that are associated with increased plasma LPS levels (Cani et al. 2007; Mohammad & Thiemermann 2021; Beam et al. 2021; Jayashree et al. 2014; Wiedermann et al. 1999).

### Peripheral endotoxin can increase amyloid pathology in mice

Lee et al. 2008 and Choi et al. 2012 reported that repeated intraperitoneal injections of LPS (250 μg/kg, 3 or 7 times in mice) resulted in Aβ accumulation in hippocampus and cortex via increased APP expression and increased β- and γ-secretase activities, as well as memory impairment. Intraperitoneal LPS increased expression of brain APP, soluble Αβ and diffuse plaques (Sheng et al. 2003; Wang et al., 2018; Wang 2018).

Transgenic mice with amyloid pathology (due to expression of mutant human amyloid precursor protein, APP) had increased proinflammatory cytokine expression in the brain when injected intraperitoneally with LPS (0.3 mg/kg), relative to wild-type mice, indicating a synergy between amyloid and LPS in inducing neuroinflammation (Knopp et al. 2022). Aβ can facilitate LPS uptake into neurons, where LPS apparently disrupts gene expression (Lukiw et al. 2019). Zhou et al. (2019) reported that intraperitoneal LPS increased microglial activation, induced neuronal apoptosis and aggravated cognitive impairment in APP/PS1 mice. Systemic LPS (1 mg/kg i.p.) also increased microglial activation and reduced microglial clearance of amyloid beta in APP/PS1 mice (Tejera et al. 2019). Systemic LPS also caused synaptic loss and memory deficits specifically in aged APP/PS1 mice (Beyer et al. 2020). Intraperitoneal injection of high dose LPS (3 mg/kg) disrupts the blood-brain barrier (BBB) of mice, and increased brain influx and decreased brain efflux of Aβ, potentially promoting Aβ accumulation in brain (Jaeger et al. 2009). And mice with amyloid plaques were more susceptible to LPS-induced opening of the BBB (Barton et al. 2019). Note, however, that injection of endotoxin into rodent brain can reduce amyloid plaques, apparently by inducing microglia to phagocytose amyloid (Mizobuchi & Soma 2021).

Aβ itself is antimicrobial against a range of clinically relevant microorganisms, including Gram-negative bacteria, and the Aβ levels found in AD brain are sufficient to block growth of bacteria such as *E. coli* (Soscia et al. 2010). This leads to the idea that brain infections (or signs of infection such as LPS) stimulate Aβ production and aggregation (and this stimulates inflammation and phagocytosis) to halt such infections, but if this results in chronic inflammation over years and decades, it may also promote neurodegeneration (Moir et al. 2018). Prior to AD onset, soluble Aβ levels decline in the brain due to aggregation into fibrillar plaques (Scheltens et al. 2021), so it is possible that the loss of antimicrobial Aβ allows proliferation of microbes, including bacteria producing LPS, but this is speculation.

Ishida et al. (2017) reported that infecting the oral cavity of APP transgenic mice with *Porphyromonas gingivalis* increased blood and brain LPS levels, increased Aβ deposition and increased levels of IL-1β and TNF-α in the brain. Ding et al. (2018) and Ilievski et al. 2018 reported that infecting the oral cavity of wild-type mice with *Porphyromonas gingivalis*, resulted in *Porphyromonas gingivalis* within the brain and brain cells, increased levels of IL-1β and TNF-α in the brain, increased Aβ levels, increased tau phosphorylation, induced neuronal death and loss, and impaired learning and memory measured by water maze. Giridharan et al. (2023) found that gut puncture to induce sepsis increased amyloid plaques and cognitive decline in APP transgenic mice.

**Fig. 3:**
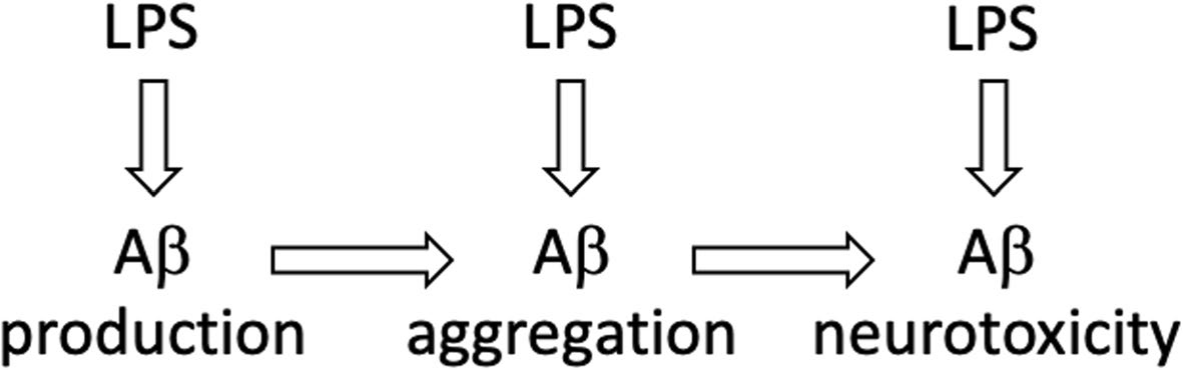
LPS increases amyloid pathology at multiple points

Reducing bacteria in the gut with broad-spectrum antibiotics reduced plaque load and microglial activation in an amyloid mouse model of AD, suggesting the possibility that LPS from the gut promotes plaque deposition and microglial activation (Minter et al. 2016; Dodiya et al. 2019). In the 5xFAD amyloid model of AD in mice, raising the mice in germ-free conditions reduced neuronal loss (relative to ‘dirty’ condition with bacteria present), and this was reversed by intraperitoneal injections of LPS (Ganz et al. 2022). This is important evidence suggesting that LPS from bacteria may promote amyloid-induced neurodegeneration in vivo. Note, however, that amyloidosis itself does not lead to neurodegeneration in such mouse models of amyloidosis (Ganz et al. 2022), and LPS can induce neurodegeneration in the absence of amyloidosis (Manabe et al., 2021), although amyloidosis increases LPS-induced neuronal loss (Ganz et al. 2022). Overall, it appears that LPS can promote amyloid pathology at the levels of Aβ production, Aβ aggregation and Aβ neurotoxicity (Fig. 3).

### Peripheral endotoxin can increase tauopathology in mice

Intraperitoneal LPS increases phosphorylation of tau protein in the brains of wild-type mice (Liu et al. 2016; Wang et al. 2018b) and in 3× transgenic AD mice (Kitazawa et al. 2005; Sy et al. 2011). This may be mediated by activated microglia releasing interleukins that activate a variety of kinases in neurons to hyper-phosphorylate tau (Li et al. 2003; Quintanilla et al. 2004; Ghosh et al. 2013; Ojala et al. 2008; Ising et al. 2019). A role of microglia is also suggested by the finding that knockout of the microglia-specific fractalkine receptor CX3CR1 increases LPS-induced tau phosphorylation, aggregation, neuroinflammation and cognitive impairment in an hTau mouse model of tauopathy (Bhaskar et al. 2010). Furthermore, knockout of mouse tau reduces LPS induced neurodegeneration and behavioural deficits in CX3CR1 knockout mice, indicating that tau is required for LPS-induced neurotoxicity (Maphis et al. 2015). Importantly, peripheral LPS can greatly increase Tau spreading in the brain in mouse models, apparently be disrupting the blood-brain barrier (Liu et al. 2020).

**Fig. 4:**
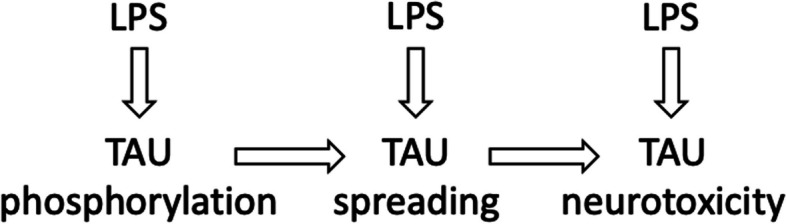
LPS increase TAU pathology at multiple points

In a mouse model of tauopathy, eliminating gut bacteria with antibiotics or germ-free upbringing, reduced microglial activation, tau pathology, and neurodegeneration in an APOE isoform-dependent manner (Seo et al. 2023). This is direct evidence that gut bacteria contribute to tauopathy and neurodegeneration, and the most obvious mediator of this effect is LPS, although other mediators have been suggested. Overall, it appears that LPS can promote tau pathology at the levels of tau phosphorylation, tau spreading and tau neurotoxicity (Fig. 4).

### Peripheral endotoxin induces loss of brain synapses, neurons and cognition in mice

Several groups have shown that intraperitoneal injection of LPS causes loss of brain synapses two months later, potentially via microglial phagocytosis of these synapses (Kondo et al. 2011; Weberpals et al. 2009; Manabe et al. 2021). Interestingly, genetic knockout or pharmacological inhibition of inducible nitric oxide synthase (iNOS/NOS2) reduced the LPS-induced microglial activation, synaptic loss and memory deficits (Weberpals et al. 2009). Cao et al. (2021) reported that i.p. injection of 50 μg/kg LPS in postnatal day (P) 14 mice resulted in microglial phagocytosis of brain synapses at P45 if the mice were stressed at that time, indicating ‘priming’ of the microglia. Ahmad et al. (2019) reported that repeated intraperitoneal injection of LPS (0.25 mg/kg/day for 7 days) strongly reduced hippocampal synapses measured by western blots 7 days later. Wu et al. (2023) used a similar model, and found loss of hippocampal synapses due to C1q-dependent microglial phagocytosis of synapses, together with cognitive impairments detected by Y maze and novel object recognition testing. Note, however, that the extent of synapse loss and memory impairment varies with LPS species and serotype, and increases in aged mice (Beyer et al. 2020).

Semmler et al. (2007) reported that 3 months after recovery from 10 mg/kg LPS injected i.p. rats had neuronal loss in hippocampus and prefrontal cortex and reduced cholinergic innervation of parietal cortex, associated with memory deficits.

Peripheral LPS can also reduce cognition in mice. Shaw KN & 2001 reported that intraperitoneal injection of 0.1mg/ml LPS impaired learning and memory of spatial information as measured by the water maze test. Lee et al. (2008), Choi et al. (2012) and Ahmad et al. (2019) reported that repeated intraperitoneal injection of LPS (0.25 mg/kg/day for 7 days) into mice impaired memory measured by passive avoidance, Y maze and water maze, measured 7 days later. Zhao et al., 2019 reported that LPS (injected i.p. at 0.5 or 0.75 mg/kg for 7 days or injected once i.c.v.) induced memory impairment in mice, measured by water maze and passive avoidance test, accompanied by microglia activation and synaptic cell loss in the hippocampus. Semmler et al. (2007) reported that 3 months after recovery from 10 mg/kg LPS injected i.p. rats had memory deficits measured by radial maze and behavioural change measured by open field, associated with neuronal loss. Hao et al. (2010) reported that rats exposed prenatally to LPS subsequently had memory deficits, measured by water maze.

Intraperitoneal injection of high dose LPS (3 mg/kg) disrupts the blood-brain barrier of mice (Jaeger et al. 2009; Barton et al. 2019). Disruption of the blood-brain barrier by plasma endotoxin potentially allows plasma endotoxin, cytokines, albumin and other factors to induce inflammation in the brain. Note, however, that this level of endotoxin is very high, and unlikely to be reached in humans other than with bacteraemia. Peripheral LPS may also indirectly affect brain function via activation of peripheral immune cells and the subsequent cytokine storm.

**Fig. 5:**
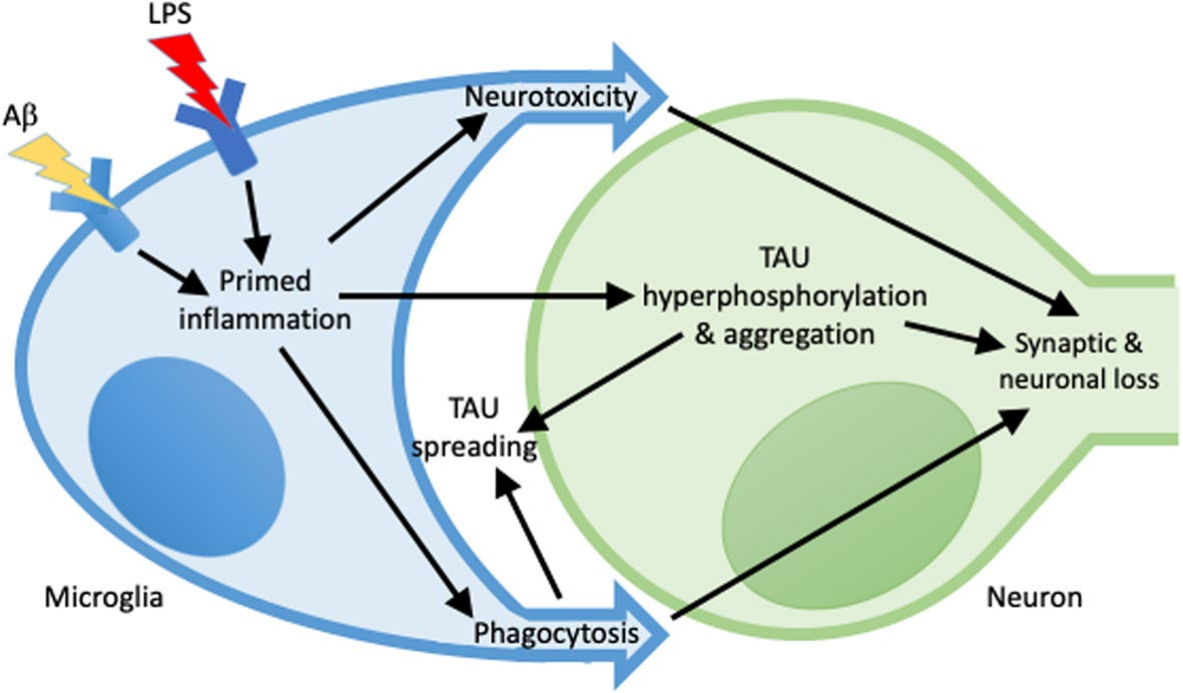
Microglia may mediate the synergy between LPS, Aβ and TAU to induce neurodegeneration. Exposure of microglia to Aβ or LPS can prime microglia, such that subsequent exposure to LPS or Aβ results in a different quantity or quality of inflammation. These inflamed microglia may induce: i) hyperphosphorylation and aggregation of TAU in neurons, ii) phagocytosis of stressed synapses and neurons, and iii) neurotoxicity. All of these may promote TAU spreading to other brain regions

Much of the neurotoxicity of LPS in mice is known to be mediated by microglia (Fig. 5), via inducing either: microglial phagocytosis of synapses (Kondo et al., 2011; Weberpals et al., 2009; Manabe et al. 2021; Cao et al. 2021; Wu et al. 2023), or phagocytosis of neurons (Neher et al. 2011; Neher et al. 2014; Milde et al. 2021), or the neurotoxicity of microglial reactive oxygen or nitrogen species (Mander & Brown 2005; Qin et al. 2013), or TAU hyperphosphorylation and aggregation in neurons (Li et al. 2003; Quintanilla et al. 2004; Ojala et al. 2008; Bhaskar et al. 2010; Ghosh et al. 2013; Maphis et al. 2015; Ising et al. 2019). Microglia are known to specifically phagocytose neurons with TAU aggregates when alive, resulting in neuronal death and TAU spreading (Brelstaff et al. 2018), and inhibition of microglial phagocytosis can reduce tauopathy in mice (Puigdellívol et al. 2021).

### Endotoxin impairs memory and mood in humans, and may induce neurodegeneration

Intravenous injection of 0.8 ng LPS/kg into healthy human volunteers impaired declarative memory for at least 9 hours (Cohen et al. 2003; Grigoleit et al. 2011). Cohen et al. 2003 reported that 0.8 ng LPS/kg improved working memory, while Reichenberg et al. (2001) reported that this increased the speed but not accuracy of working memory, while long-term memory for emotional stimuli was selectively impaired by 0.4 ng LPS/kg. However, Krabbe et al. (2005) and Grigoleit et al. 2010 found no significant effects of 0.2 ng and 0.4 LPS/kg on memory.

Intravenous injection of 0.8 or 1 ng LPS/kg into healthy human volunteers increased anxiety and depressed mood (Reichenberg et al. 2001; Grigoleit et al. 2011), and decreased motivation (alertness, energy, focus, social interest) for several hours after injection (Sandiego et al. 2015). Overall, these results indicate that intravenous injection of 0.8-1.0 ng LPS/kg into healthy human volunteers induces anxiety and depressed mood and impairs declarative memory. These symptoms overlap with AD, so it is of interest to compare the levels of LPS found in AD. As summarised above, average plasma levels of endotoxin found in AD patients were 46 ± 3 pg/ml in one study (Loffredo et al. 2020) and 60 ± 12 pg/ml in another (Zhang et al. 2009), and these levels were 3-6 times the levels of endotoxin found in age-matched controls (Zhang et al. 2009; Loffredo et al. 2020; Andreadou et al. 2021). 1 ng/kg LPS is 70 ng LPS for a 70 kg person, which is 70 ng/5l = 14 pg/ml when evenly distributed in 5 l blood volume. Thus, the levels of LPS found in the blood of AD patients apparently cause significant acute effects on memory and mood when injected into healthy volunteers. However, LPS induces a reduced sensitivity to subsequent LPS exposure (Bahador & Cross 2007), so it is difficult to compare the effects of acute and chronic exposure to LPS.

Intravenous injection of endotoxin in human volunteers is limited by ethical considerations to low doses and short duration. To examine the effects of higher doses and durations, we need to consider pathologies associated with endotoxin. “Sepsis-associated encephalopathy” is the name for the brain dysfunction associated with sepsis in humans (without any brain infection), and occurs in up to 70% of patients with sepsis i.e. severe systemic infection (Barichello et al. 2005; Comin et al. 2009, Widmann & Heneka 2014). Symptoms are variable, but typically include delirium, associated with hallucinations, restlessness or agitation, during sepsis, but also long-term cognitive and emotional impairment after sepsis. Most (but not all) cases of sepsis are caused by gram-negative bacteria in the blood, and in these cases much of the pathology is attributed to endotoxin in the blood. Thus, it would appear that high doses of LPS in the blood for several days can cause long-term cognitive deficits and symptoms that overlap with AD.

Hepatic encephalopathy results in chronically elevated endotoxin in blood, similar to endotoxin levels found in AD for months or years, and the encephalopathy has been attributed to the elevated endotoxin or ammonia (Jain et al. 2012). Early symptoms of hepatic encephalopathy are: confusion, forgetfulness, personality or mood changes, poor judgement, poor concentration and change in sleep pattern. Late and severe symptoms include: hands or arms movements, extreme anxiety, seizures, severe confusion, sleepiness or fatigue, severe personality changes, jumbled and slurred speech and slow movement. Some of these symptoms overlap with those of AD, so if the symptoms of hepatic encephalopathy are due to elevated endotoxin, then the overlapping symptoms of AD could also be due to elevated endotoxin.

### Limitations and evidence potentially contradicting the endotoxin hypothesis

It is important also to discuss the limitations of the endotoxin hypothesis. Mouse and rat studies have been used to support the endotoxin hypothesis. However, rodents are much less sensitive to LPS than humans, and have higher plasma levels of LPS, and so the human relevance of rodent models is unclear (Bahador & Cross 2007).

Moreover, the response to LPS depends on the concentration and time course of administration as prior exposure to LPS can either sensitise or desensitise to subsequent LPS or pathology (Bahador & Cross 2007; Wendeln et al. 2018). For example, in an amyloid mouse model of AD, a single intraperitoneal injection of LPS (0.5 mg/kg), which sensitised the brain to subsequent LPS, increased amyloid plaques and associated neuritic damage 6 months later, but 4 such injections on 4 successive days, which desensitised the brain to LPS, reduced amyloid plaques and associated neuritic damage 6 months later (Wendeln et al. 2018). Similarly, in amyloid mouse models, intrahippocampal injection of LPS reduced total Aβ level, without affecting amyloid plaque load, potentially by activating glia to remove Aβ (Dicarlo et al. 2001; Herber et al. 2004). Low dose LPS has been found to be neuroprotective in other animal models of AD (Mizobuchi & Soma 2021). For example, low-dose endotoxin (0.15 mg/kg weekly for 3 months) given to transgenic mice overexpressing human Tau mutant (P301S) in neurons, reduced TAU phosphorylation and improved cognitive function, apparently by increasing neuronal autophagy. Thus, it would appear that LPS has a variety of effects, some beneficial and some detrimental in mouse models of AD; however, the majority of such studies report detrimental effects of LPS.

Increased blood levels of LPS are not specific to AD, but also found in sepsis, periodontitis, liver disease, diabetes, amyotrophic lateral sclerosis and Parkinson’s disease (Brown 2019). So, LPS can’t be sufficient to induce AD. Something else must be required for AD, for example, LPS plus amyloid and/or tau pathology, or LPS plus APOE4. Note that amyloid and tau aggregation are also not specific to AD, but occur in multiple neurodegenerative diseases. Neurodegeneration might prime the brain for an excessive LPS response, or prior exposure to LPS may prime the brain to a subsequent neurodegenerative process (Cunningham 2013; Neher & Cunningham 2019). It also seems that increased blood LPS is not required for AD, because blood LPS is only increased in some AD patients (Fig. 2). However, it is possible that LPS exposure prior to neurodegeneration may sensitise to neurodegeneration much later, when blood LPS is low (Wendeln et al. 2018).

### Conclusions, key tests and potential treatments based on the endotoxin hypothesis

How might endotoxin, Aβ and Tau interact to cause AD? AD pathogenesis is generally thought to have three steps: i) Aβ aggregation, ii) microglial activation, and iii) Tau aggregation and spreading, resulting in synaptic and neuronal loss. As outlined above, endotoxin might act at each of these three steps inducing: i) Aβ production, seeding and aggregation, ii) microglial priming and activation, and iii) Tau phosphorylation and spreading; and each of these three processes can cause synaptic and neuronal loss (Figs. 1, 3, 4 and 5).

The endotoxin hypothesis of AD suggests that elevated LPS levels contribute to the pathogenesis of AD. In animal models of AD, it would be useful to test whether reducing LPS or LPS response (e.g. by the treatments suggested above) can reduce pathology. However, the key test of this hypotheses is whether reducing LPS (for example by the treatments suggested below), in those AD patients with increased LPS, can reduce the rate of disease progression.

If the endotoxin hypothesis of AD is correct, then a variety of potential treatments might be tried for AD, designed to either lower blood LPS levels or the response to LPS, including those listed below. Treatments designed to lower blood LPS should be targeted at AD or MCI patients with elevated blood LPS levels, and ideally designed to reverse whatever is causing this), and efficacy verified by measuring blood LPS levels longitudinally, as well as AD disease progression. Some treatments designed to lower blood LPS or LPS response are listed below.

1. Gut microbiome. Changing the gut microbiome to reduce inflammatory LPS-producing bacterial species, or increase anti-inflammatory species, can be done with: specific antibiotics, oral bacteria or faecal transplant (Mohammad & Thiemermann 2021).
2. Gut permeability. Treatments to reduce gut permeability may include: a) upregulation of mucin-producing bacteria or reduction of mucin-degrading bacteria (Mohammad & Thiemermann 2021), b) anti-TNF-α antibodies to reducing gut inflammation (Suenaert et al. 2002), or c) metformin (Ahmadi et al. 2020).
3. Infections. Gram-negative bacterial infections of the gut, gums, lungs or urinary tract can be sources of elevated blood LPS, and can be treated with antibiotics or preventative measures (Tate et al. 2014; Fu et al. 2023).
4. Blood LPS. Vaccination with non-toxic forms of LPS can in principle induce anti-LPS antibodies to lower blood LPS levels (Cross et al. 2014).
5. Response to LPS. The microglial and other cellular responses to LPS (or inflammation induced by LPS) can in principle be reduced by blocking: a) TLR4 (Rice et al. 2010), b) complement activation or complement receptor 3 (Bodea et al. 2014; Wu et al. 2023), c) the P2Y6 receptor (Milde et al. 2021; Puigdellívol et al. 2021) or d) the inflammasome (Heneka et al. 2018; Chen et al. 2020). However, blocking TLR4 or these other targets may reduce innate immunity, so it is at present unclear whether this would be of net benefit in AD.

## Data Availability

Only publicly available data is evaluated in this manuscript.

## Abbreviations

Aβ: Amyloid beta
AD: Alzheimer’s disease
APOE: Apolipoprotein E
APP: Amyloid precursor protein
BIN1: Bridging integrator-1
CLU: Clusterin
CR1: Complement receptor 1
CSF: Cerebrospinal fluid
CX3CR1: CX3C motif chemokine receptor 1
ELISA: Enzyme-linked immunosorbent assay
IL-1β: Interleukin 1β
IL-6: Interleukin 6
iNOS: Inducible nitric oxide synthase
LAL: Limulus amebocyte lysate
LOAD: Late-onset Alzheimer’s disease
LPS: Lipopolysaccharide
MCI: mild cognitive impairment
MD2: Myeloid Differentiation factor 2
TLR4: Toll-like receptor 4
TNF-α: Tumor necrosis factor α
TREM2: Triggering receptor expressed on myeloid cells 2
